# Statistical Properties Demand as Much Attention as Object Features

**DOI:** 10.1371/journal.pone.0131191

**Published:** 2015-08-21

**Authors:** Liqiang Huang

**Affiliations:** Department of Psychology, The Chinese University of Hong Kong, Hong Kong, China; University of Bath, UNITED KINGDOM

## Abstract

Recent studies have argued that the statistical properties of a set of visual items can be extracted with little or even no cost. In the present study, observers (*N* = 188) performed a color task and an orientation task, and the attention effect was measured as the advantage of pre-cueing one of the two tasks. The color and orientation tasks required participants to report either an object feature or the mean of a 4×4 array (i.e., statistical property). The pre-cueing advantages were approximately equal regardless of the nature of the tasks (object features vs. statistical properties), providing evidence that statistical properties are not perceived with zero cost, but demand as much attention as object features.

## Introduction

Studies of visual attention have usually used objects that are homogenous in terms of their features: For example, a red object is homogenously red across the whole object. However, real-world objects are often heterogeneous. For example, a so-called yellow tree may also include reddish leaves and greenish leaves. Yet, when looking at this tree, one perceives the “overall color” of the tree quickly and effortlessly.

For ease of description, in this study, the term “statistical properties” refers to the statistical properties of a heterogeneous set, whereas the term “object features” refers to the features of a homogenous object. Certainly, the use of different terms does not intend to imply that statistical properties are orthogonal to the object features. After all, a statistical property (e.g., average size of a set) is a statistical summary of the object features (e.g., object size) of all the elements in a set of objects.

### Previous Findings

Studies of statistical properties have been an important trend in the field of attention and perception in the last decade (see [1] for a review). Researchers have shown that statistical properties can be extracted efficiently [2–8], that they systematically affect the ways in which information is encoded [9–10], and that they play an important role even in high-level visual functions such as the perception of gaze directions [11] or facial attractiveness [12].

One of the most important claims made about statistical properties is that their attentional demand is low [3–8]. However, previous studies have often only given implicit, and sometimes inconsistent, indications of what should be considered “normal” attentional demand. Below, I will illustrate two separate concepts that have been associated with the notion of the *low attentional demands* of statistical properties.

### Two Concepts of Statistical Properties

A first concept is *set-level encoding*, namely that statistical properties are perceived directly on the level of a set rather than on the level of an individual element. This implies that the statistical summary of a set can be directly perceived even if individual features in the set remain unreportable. In a sense, set-level encoding can be interpreted as evidence for *low attentional demand*. When observers are asked to report a statistical summary of a set, they can do significantly better than what would have been expected if they could only serially scan the individual elements of the set and speculate on the value of the summary on the basis of individual values. There is abundant evidence in favor of *set-level encoding* [2–7].

Another concept that has sometimes been associated with the low attentional demand of statistical properties is *cost-free encoding*, namely that perceiving statistical properties is a “cost-free” process. For example, a previous study argued that it takes less attention to perceive statistical properties [6], and another study made a more explicit claim that it is “cost-free” to perceive the statistical summary of stimuli that are out of attentional focus [8]. In these studies, the critical evidence for *cost-free encoding* is that the performance of a central task remained constant regardless of whether observers had to additionally report the statistical summary of items that were out of attentional focus.

Set-level encoding and cost-free encoding are clearly two separable concepts. *Set-level encoding* merely implies that statistical property can be perceptually encoded. *Cost-free encoding* makes a much stronger claim that this encoding is cost-free.

### Attentional Cost in the Encoding of Statistical Properties

Although set-level encoding is well established, it still seems premature to accept the notion of cost-free encoding. For one thing, researchers have generally argued that attention is the mechanism that allows us to consciously perceive things [13–15]. In other words, no visual information of any kind can be perceived without an attentional cost. There is an apparent conflict between this very general rule and the findings of cost-free encoding of statistical properties. This study attempts to shed light on this conflict.

**Fig 1:**
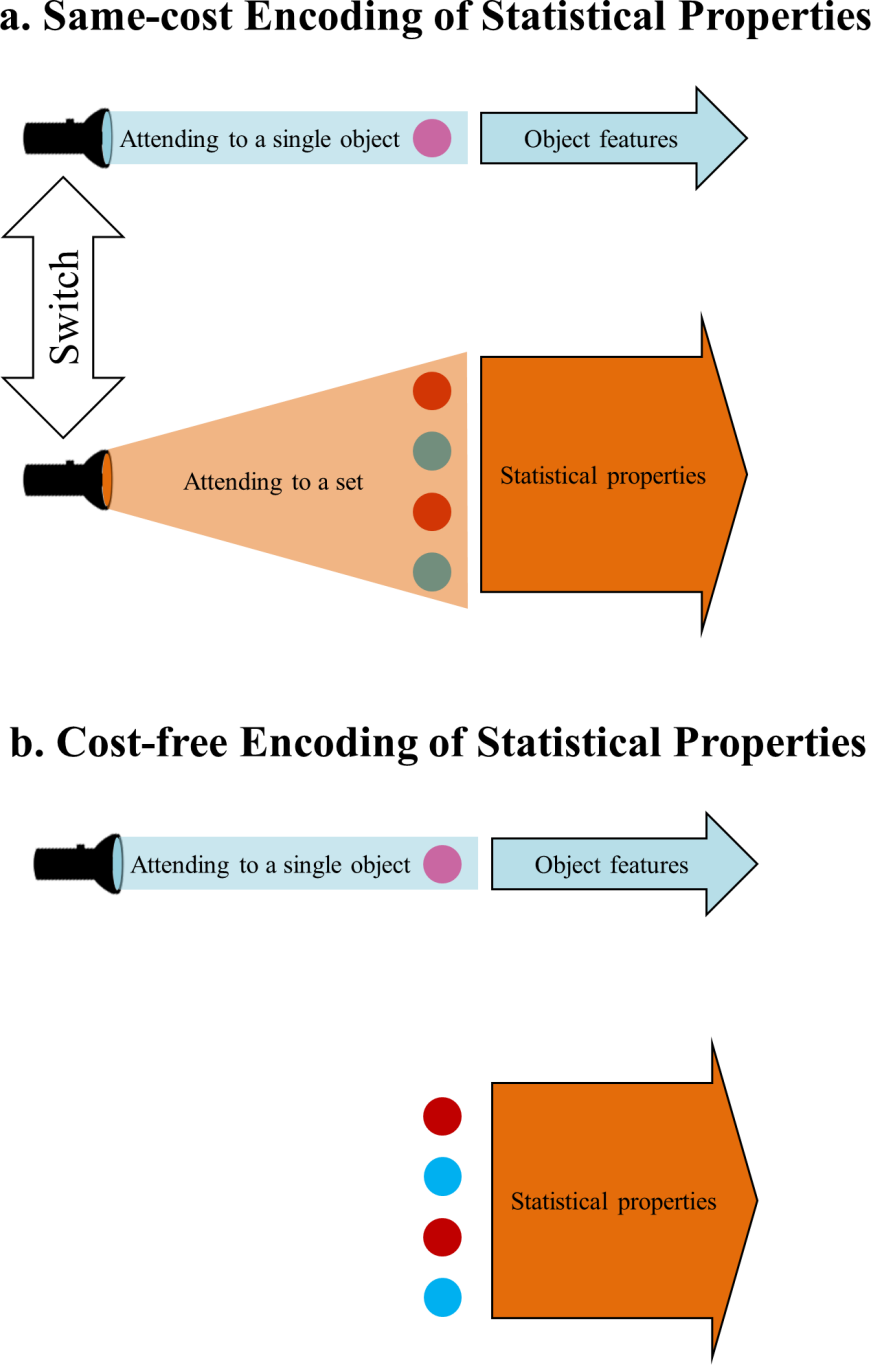
Two possible accounts for the attentional demand of statistical properties. Panel A illustrates the same-cost account of the encoding of statistical properties: Attention can be focused on a single object or distributed over a set, and it allows observers to perceive the attended information in essentially the same way, no matter whether this information is an object feature or statistical property. Panel B illustrates the cost-free account of the encoding of statistical properties: Attending to a single object allows observers to perceive its features, whereas the statistical properties of a set that is out of attentional focus can be simultaneously perceived for free without any additional cost.

For ease of description, Fig 1 gives two possible accounts of the attentional cost of statistical properties. A *same-cost account* holds that the general attentional cost for conscious perception also applies to the encoding of statistical properties. In contrast, a *cost-free account* holds that there is no attentional cost for encoding of statistical properties.

We need a baseline for a “general cost” so that we can know, experimentally, whether the observed attentional cost of statistical properties is closer to this general cost or to zero (i.e., no cost). This study uses object features as this baseline. There are two reasons for this choice. First, object features are the more typical stimuli in the broader literature. Second and more importantly, statistical properties are by definition special in that they belong to a set rather than a single object. So, conceptually speaking, object features are the default baselines if one wants to claim that the statistical properties are special.

After determining the baseline, the two accounts can be further specified as follows. In the *same-cost account*, attention can be focused on a single object or distributed over a set, and it allows observers to perceive the attended information, no matter whether that information is an object feature or statistical property, in essentially the same way. In the *cost-free account*, attending to a single object allows observers to perceive its features, whereas the statistical properties of a set that is out of attentional focus can be simultaneously perceived for free without any additional cost.

### The Present Approach

In this study, a color stimulus and an orientation stimulus, each of which could independently be either an object feature or the statistical property of a heterogeneous set, were simultaneously presented. The attentional demand of these tasks was operationalized as a standard pre-cueing advantage. In the pre-cue condition—but not the post-cue condition—the observers were told in advance which of the two tasks (i.e., color vs. orientation) would be tested. Therefore, the observers could only focus on the target in the pre-cue condition, but they had to try to perceive both stimuli in the post-cue condition. The magnitude of this pre-cueing advantage (advantage in the pre-cue over the post-cue condition) was then used as a measure of the attentional demand of the task.

If the same-cost account is correct, then we would expect to see significant and comparable pre-cueing advantage in all conditions. If the cost-free account is correct, then we would expect to see no pre-cueing advantage when either the color or orientation task is about statistical properties and a substantial pre-cueing advantage only when both tasks are about object features. These predictions will be compared with the results of the present experiment.

## Experiment

### Method

#### Ethics statement

This study received prior ethical approval from research ethics committee of the Chinese University of Hong Kong. The committee approved the consent form and experimental procedure. Written consent was obtained from the participants.

#### Participants

University undergraduate students, all of whom had normal or corrected-to-normal vision, participated in this study. Twelve participants were excluded because their performance was beyond a predetermined criterion. Specifically, eleven participants were excluded because their performance was close to the floor (<0.6), and one participant was excluded because her performance was close to the ceiling (>0.9). Including these participants in the analysis does not change the conclusion.

Aside from these excluded participants, a total of 188 participants took part in the study. This sample size was determined in advance on the basis of pilot testing of similar designs. The sample size was unusually large for two reasons. First, I wanted to make within-subject comparisons for as many as 16 conditions (see below). Second, the same-cost account was tentatively supported in a pilot experiment. Therefore, the present experiment was designed with the expectation of confirming a lack of differences, an outcome that is known to be statistically demanding.

#### Apparatus

In both experiments, the stimuli were presented on a 1,024 × 768 pixel CRT monitor and the participants viewed the display from a distance of about 60 cm. The participants responded by pressing one of two adjacent keys (“j” or “k”).

#### Stimuli and Procedure

**Fig 2:**
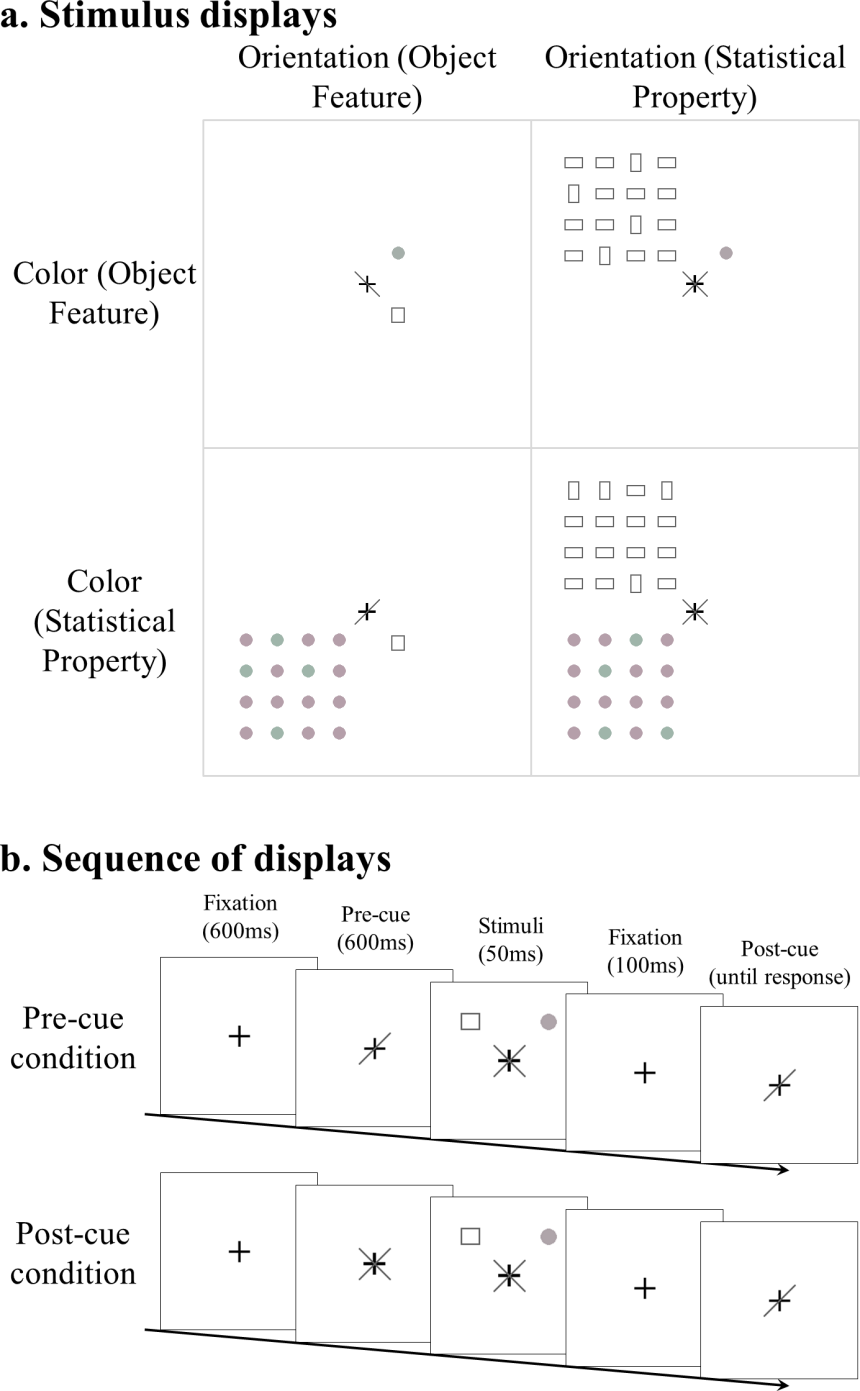
Method. A. Stimulus display. This stimulus display consisted of a rectangle (or a 4×4 array of rectangles) in the top-left or bottom-right quadrant and a circle (or an array of circles) in the top-right or bottom-left quadrant. The circle (or the array of circles) could be reddish or greenish. The rectangle (or the array of rectangles) could be vertical or horizontal. When an array was reddish (or vertical), this just meant that 12 of the 16 items in the array were reddish (or vertical). The participants were asked to report one of these features, as specified by the post-cue. B. Sequence of display. The stimulus display was preceded by a pre-cue and followed by a post-cue. The post-cue always specified the target, whereas the pre-cue specified the target in half of the trials (pre-cue condition) but not in the other half (post-cue condition).

The sequences of the presentations are shown in Fig 2B. A trial started with a black fixation cross, which was presented in the center of the display for 600 ms. This was followed by a pre-cue display lasting 600 ms, after which the stimulus display was presented. The stimulus display was presented for 50 ms and then disappeared. It was followed by a blank display lasting 100 ms, after which the post-cue display was presented. The post-cue remained on the screen until a response was made.

As shown in Fig 2A, a stimulus display consisted of a rectangle (or a 4×4 array of rectangles) in the top-left or bottom-right quadrant and a circle (or an array of circles) in the top-right or bottom-left quadrant. The circle (or the array of circles) could be reddish or greenish. The rectangle (or the array of rectangles) could be vertical or horizontal. When an array was reddish (or vertical), this just meant that 12 of the 16 items in the array were reddish (or vertical). The featural differences were made much subtler for stand-alone items than for arrays so that the task difficulties were approximately matched. Altogether, there were four types of display (Fig 2A): Color (Object Feature)—Orientation (Object Feature); Color (Object Feature)—Orientation (Statistical Property); Color (Statistical Property)—Orientation (Object Feature); Color (Statistical Property)—Orientation (Statistical Property).

The participants were asked to report the feature of the target item or target array. This feature was specified by a post-cue (i.e., a left-tilted or right-tilted black bar). If a target was vertical or reddish, they pressed “j”; if a target was horizontal or greenish, they pressed “k.” They were asked to respond as accurately as possible but were under no time pressure (i.e., unspeeded responses).

In half of the trials, the target was specified in the pre-cue display so that the participants could focus on the target only. In the other half, the pre-cue was given as an uninformative cross so that the participants had to perceive all of the visual stimuli.

In sum, there were four types of displays (see above) × two types of cues (pre-cue vs. post-cue) × two types of tasks (color task vs. orientation task), making 16 conditions in total. These conditions were all mixed within each block. Each participant completed nine blocks (80 trials per block). The first block was treated as practice and excluded from the analysis.

### Results

**Fig 3:**
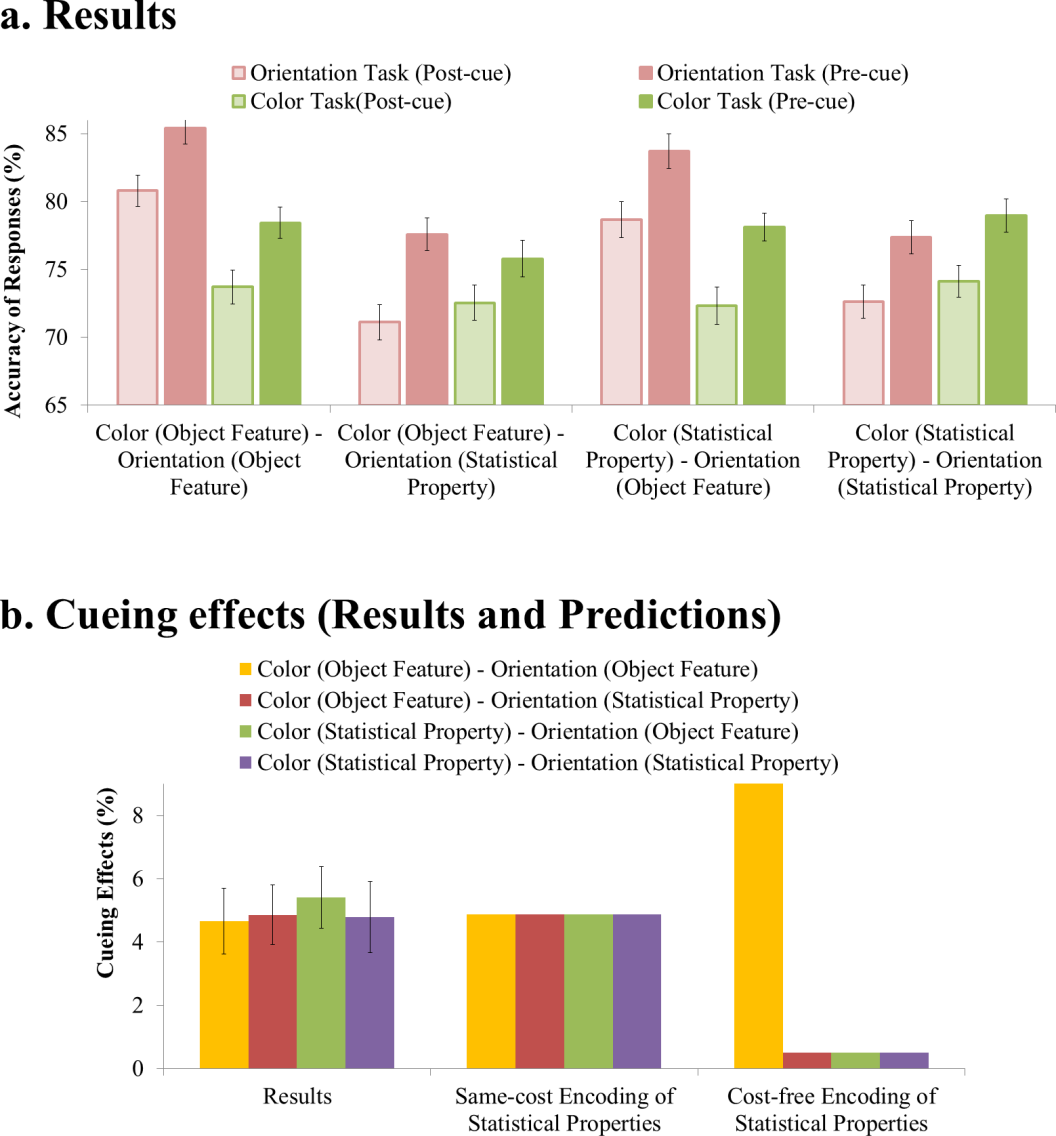
Results. Panel A shows the observers’ accuracies in all 16 conditions. There were significant pre-cueing advantages for both the color and orientation tasks regardless of whether each task was about object features or statistical properties. Panel B shows the average pre-cueing advantages for the color and orientation tasks. The predictions are given to illustrate the predicted directions of effects and are not meant to be numerically precise. It is clear that the results of the experiment are consistent with the same-cost account of the encoding of statistical properties and inconsistent with the cost-free account of the encoding of statistical properties. Error bars show within-subject 95% confidence intervals [22].

The observers’ accuracies in all 16 conditions are presented in Fig 3A. There were significant pre-cueing advantages for both the color and orientation tasks regardless of whether the task was about object features or statistical properties (*p* < .000005; *Cohen’s d* > 0.33 in all 8 pairs of comparisons). Individual-level data from all participants can be found in S1 Data.

More importantly for the present purposes, Fig 3B presents the average pre-cueing advantages for the color and orientation tasks along with the predictions of the two accounts given above. The predictions are only given to illustrate the predicted directions of effects and are not meant to be numerically precise. It is clear that the results are consistent with the same-cost account and inconsistent with the cost-free account: There were significant pre-cueing advantages in all conditions in which statistical properties were required in the task, and these pre-cueing advantages were no less than in the condition in which two object features were required.

## General Discussion

This study systematically compared the attentional demand of statistical properties and object features. The results clearly demonstrated attentional costs of encoding statistical properties and challenge the view that it is a cost-free process. In addition to the experimental support from these results, the same-cost account is also appealing because of its conceptual simplicity and ecological validity. Visual stimuli in the real-world environment seem to constitute a continuum that peaks at moderate heterogeneity rather than a dichotomy between perfectly homogenous objects and very heterogeneous sets. Therefore, it seems implausible that the visual system would develop two separate strategies/mechanisms.

Naturally, the present results cannot rule out the possibility that attentional costs are slightly lower in encoding statistical properties than object features. Nevertheless, given the fairly large sample size and fairly precise measurements of the pre-cueing advantages, it seems safe to say that any such difference, if it exists, must be trivial in magnitude.

This study only focused on attentional competition between tasks, for which purpose the pre-cueing paradigm is a sensible test. Therefore, the present conclusion is not necessarily inconsistent with the previous claims on the uniqueness of statistical properties in other designs such as object substitution masking [16] and attentional blink [17]. Future studies will be needed to better integrate these findings.

### Cost-free Statistical Properties Reconsidered

How then can we explain the previous findings of cost-free statistical properties [6, 8]? I argue that these studies underestimated the attentional costs because they used unbalanced stimuli. These previous studies have generally shown that a first task of reporting target features is not impaired by an additional task of reporting the statistical properties of the “background stimuli.” However, in these experiments, the first task always appeared to be a “primary” task. Therefore, it seems plausible that the participants in these studies tried to give as much attention as possible to the first task. This explains why the performance was equal regardless of the additional task.

On the other hand, it is well known that attention often cannot be fully allocated to the intended task and that there will be some “spillover” for the processing of other on-going stimuli [18]. This explains the better-than-guessing performance in the additional task. If these speculations are correct, then we would expect to see significant competition between the central task and the “statistical property” task when they become equally important. The present experiment confirmed exactly this.

Other than the cost-free account illustrated in Fig 1B, the previous findings of cost-free statistical properties [6, 8] can also be potentially explained by assuming that object features and statistical properties are governed by distinct types of attention. Specifically, there are attentional costs of encoding either object features or statistical properties, but these two types of attentional costs are independent of each other. This account differs from the cost-free account by predicting a significant pre-cueing advantage when observers simultaneously perceive two statistical properties of two sets, but this account agrees with the cost-free account on predicting no pre-cueing advantage when observers have to try to simultaneously perceive one object feature and one statistical property. Therefore, this potential account is also rejected by the present results.

### Relation to the Boolean Map Theory

The present study reconfirms the link between attention and consciousness, and it challenges the conclusion of previous studies [6, 8]. All in all, although statistical properties are encoded on the level of sets, this encoding is subject to the same general attentional limit and is not cost-free.

Both of these points (set-level encoding and same-cost encoding) are embraced by the Boolean map theory of visual attention [19–21]. In this theory, my collaborators and I proposed that all conscious perception boils down to one single data format (i.e., the Boolean map) and the controls of attention boil down to the ways of creating a Boolean map. This formalization implies that the conscious access to any information always bear the cost of one-at-a-time creation of Boolean maps and therefore predicts that encoding of statistical properties (i.e., creating a Boolean map that covers a whole set) is not cost-free. Instead, it is subject to the same attentional cost as encoding of object features (i.e., creating a Boolean map that covers a single object).

In addition, the “Boolean” nature of the Boolean map, as defined by [19], implies that the map only carries the featural information associated with the entire map (e.g., the statistical properties of the entire set), not that which belongs to only a part of the map (e.g., the features of one individual element). Therefore, when a whole set is selected by attention (i.e., creating a Boolean map that covers this set), Boolean map theory predicts a set-level encoding, rather than encoding of individual elements. All in all, the Boolean map theory offers a straightforward way of understanding the role of attention in perceiving statistical properties, which is consistent with all of the results discussed above.

## Supporting Information

S1 Data Spreadsheet containing dataset for the present manuscript. (XLSX)
